# Volatiles from Plants Induced by Multiple Aphid Attacks Promote Conidial Performance of *Lecanicillium lecanii*

**DOI:** 10.1371/journal.pone.0151844

**Published:** 2016-03-21

**Authors:** Yongwen Lin, Mubasher Hussain, Pasco Bruce Avery, Muhammad Qasim, Dalin Fang, Liande Wang

**Affiliations:** 1 College of Plant Protection, Fujian Agriculture and Forestry University, Fuzhou, China; 2 Key Laboratory of Biopesticide and Chemical Biology, Ministry of Education, Fuzhou, China; 3 Key Laboratory of Integrated Pest Management for Fujian-Taiwan Crops, Ministry of Agriculture, Fuzhou, China; 4 Indian River Research and Education Center, University of Florida, Institute of Food and Agricultural Sciences, Fort Pierce, Florida, United States of America; Fujian Agriculture and Forestry University, CHINA

## Abstract

Herbivore-induced plant volatiles (HIPVs) are clues that help predatory insects search for food. The hypothesis that entomopathogenic fungi, which protect plants, benefit from the release of HIPVs was tested. The plant *Arabidopsis thaliana* was used as the source of HIPVs. The insect herbivore *Lipaphis erysimi* (Kaltenbach) was used as the inducer, and the fungal pathogen of the aphid *Lecanicillium lecanii* was exposed to HIPVs to test our hypothesis. When exposed to aphid-induced *A*. *thaliana* volatiles, the mortality of aphids pre-treated with a conidial suspension of *L*. *lecanii*, the conidial germination and the appressorial formation were significantly increased compared with the control. The decan-3-ol and 4-methylpentyl isothiocyanate that were detected in the headspace seemed to have positive and negative affection, respectively. Moreover, HIPVs generated from groups of eight aphids per plant promoted significantly increased conidial germination and appressorial formation compared with HIPVs from groups of one, two and four aphids per plant. Our results demonstrated that the pathogenicity of the entomopathogenic fungus *L*. *lecanii* was enhanced when exposed to HIPVs and that the HIPVs were affected by the number of insect herbivores that induced them.

## Introduction

Most plants produce toxic compounds and emit herbivore-induced plant volatiles (HIPVs) for protection when attacked by various insect herbivores [1–7]. As an indirect defensive strategy of the plant, HIPVs can “alert enemies of insect herbivores for help” [7–9]. Some of the compounds emitted as HIPVs that attract or impact predator behavior have been chemically separated and analyzed in several studies. For example, the predatory mite *Neoseiulus womersleyi* exhibited a significant preference for a mixture of three compounds [(E)-β-ocimene, (E)-4,8-dimethyl-1,3,7-nonatriene, and (E,E)-α-farnesene] in tea leaves [10], whereas tachinid flies were highly attracted to cis-3-hexen-1-ol [11]. Maize that was damaged by lepidopteran pests produced (E)-β-caryophyllene from roots and leaves to attract parasitic wasps that deposit their eggs into the larvae, thus incapacitating the larvae and minimizing plant damage [12]. Methyl salicylate, a major compound emitted in HIPVs, was the key factor that attracted lacewings (*Chrysoperla externa*) to aphid-infested rose plants [13].

Natural enemies can use HIPVs to locate their hosts or prey [14]. For example, in an experiment conducted using a Y-tube olfactometer, nymphs and adults of experienced mirid bugs, *Macrolophus pygmaeus* (Rambur), were attracted by HIPVs, which were emitted from prey-infested plants [15]. In another study, some chemical components of HIPVs regulated electrophysiological characteristics and behaviors of wasps [16]. In addition, diamond back moth larvae-induced cabbage-plant volatiles were an important factor that affected the response of the parasitoid, *Cotesia vestalis*, to different colored lights [17]. HIPVs can also act as a distress signal cue from the damaged plant to the surrounding vegetation [18–20]. For example, HIPVs emitted from blueberry leaves fed upon by the gypsy moth triggered undamaged leaves to accumulate increased amounts of endogenous *cis*-jasmonic acid compared with non-exposed leaves [21], and it should be the reason that *Spodoptera exigua* were repelled by the Arabidopsis which primed with HIPVs [22]. Moreover, several resistance marker genes (PATHOGENESIS-RELATED [PR] 1, 2 and 4) in susceptible common bean (*Phaseolus vulgaris*) were activated by volatile organic compounds (VOCs) from resistant plants [23].

In addition, some research has demonstrated that microorganisms, including fungal pathogens, can be impacted by plant VOCs. *Trans*-2-hexenal and *cis*-3-hexenal, green-odor compounds emitted by the green leaves of rice plants have demonstrated a remarkable ability to suppress disease attributed to the rice blast fungus, *Magnaporthe oryzae*, under laboratory conditions [24]. More recently, VOCs induced from the common bean leaves, including limonene, linalool, nonanal, methyl-salicylate and methyl-jasmonate, directly inhibit conidial germination of the fungal pathogen *Colletotrichum lindemuthianum* [23]. The germination of the conidia of the entomopathogenic fungus *Metarhizium anisopliae* was inhibited by the VOCs emitted from highly insect-tolerant cowpea leaves [25]. The effects of HIPVs emitted from plants on entomopathogenic fungi and the potential role of HIPVs as protectants is currently being investigated [26,27]. For example, Brown et al. [28] reported that the germination of conidia of the entomophthoralean pathogen *Pandora neoaphidis* was inhibited by HIPVs induced by tobacco aphids feeding on tobacco plants. Hountondji et al. [29] demonstrated that HIPVs induced from cassava by the cassava green mite *Mononychellus tanajoa* influence conidia and capilliconidia production of the fungal entomophthoralean, *Neozygites tanajoae*. Few studies have investigated the effect of HIPVs or VOCs on *Hypocreales* [25,30]. In a literature review, Elliot et al. [26] introduced the hypothesis that plants may utilize fungal entomopathogens as protectants. The question of whether plants can increase their own fitness has not been tested in tritrophic systems and remains to be demonstrated [27]. Therefore, research investigating the effect of HIPVs on the pathogenicity of the fungal entomopathogens against any herbivore must be conducted.

A structure that is a precursor to the infection process of pathogenic fungi is the formation of the appressoria, which are swollen, dome-shaped cells [31–33]. In the presence of nutrients and water, conidia of fungi form germ tubes, and the germ tubes form appressoria on the host and non-host cuticle [34]. These structures can also be formed on the surface of glass slides and hydrophobic membranes [35,36]. When conidia are placed on these artificial surfaces, such as glass or plastic, it typically requires 36 to 40 h to complete appressorial formation [37]. However, differentiation in the rate of appressorial formation is affected by various factors, including the substrate surface and abiotic conditions (temperature, humidity, pH value, etc.) [34,35,38]. For example, Spence et al. [39] discovered that the volatile, hydrogen cyanide produced by rice rhizospheric bacteria inhibited appressoria formation in *Magnaporthe oryzae*.

In this study, we investigated two systems: 1) the HIPV emitting system in which the volatiles were induced by aphids feeding on *A*. *thaliana* plants and 2) an entomopathogenic fungal infection system in which the aphid was treated with *Lecanicillium lecanii* prior to exposure to the aphid-induced HIPVs [40,41]. To evaluate the influence of HIPVs on entomopathogenic fungi, it was hypothesized that *A*. *thaliana* would modify its VOCs and emit HIPVs when infested by aphids [42]. Lastly, we compared the performance of the conidia and appressorial formation with or without exposure to aphid-induced HIPVs. Overall, the aim of this study was to understand whether HIPVs enhance or deter the pathogenicity processes of entomopathogenic fungi-infecting herbivores.

## Materials and Methods

### Organisms

*Arabidopsis thaliana* was planted at 25°C and 75% relative humidity (RH), under a 16 h light:8 h dark (L:D) photoperiod. Forty-day-old plants were used for the experiments.

The aphid *Lipaphis erysimi* Kaltenbach was collected from cabbage fields on the Fujian Agriculture and Forestry University (FAFU) campus and bred in netted cages (50 cm × 50 cm × 50 cm) in the laboratory at 25°C, 75% RH, and a 16 h:8 h L:D photoperiod on *A*. *thaliana*. Two-day-old apterous adult aphids were used in the experiments.

The fungus *Lecanicillium lecanii* strain V3450 was isolated from *Siphoninus phillyreae* Haliday and was stored and maintained at 4°C with Czapek's medium [34] in our laboratory at FAFU until needed.

### HIPV-inducing systems and fungal infection testing protocols

**Fig 1:**
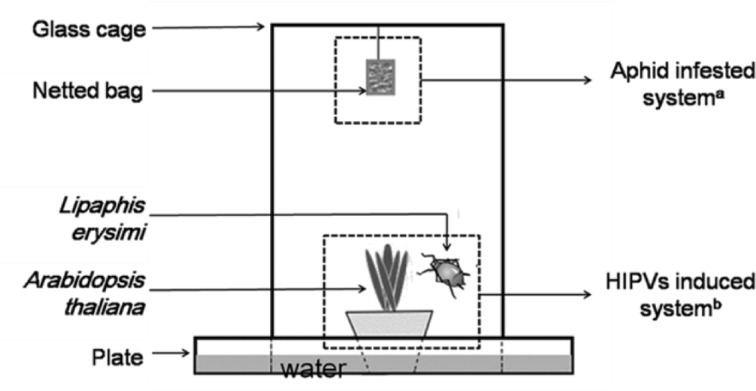
Side view of the HIPVs induced and aphid fungi-infected systems. ^a^ *Lecanicillium lecanii* infected aphids (*Lipaphis erysimi*) inside of the netted bags were suspended from the top of the cage while being exposed to HIPVs (aphid-infected system) over time. ^b^ Different numbers (0, 1, 2, 4, 8, and 16) of aphids feeding on a potted *Arabidopsis thaliana* plant inside a glass cage induced the HIPVs (HIPV-induced system).

Two systems were utilized in the experiment: the HIPV-inducing system and the fungal-infecting system. The systems were used to test the sub-lethal time (LT_50_) values (d) of the fungus for infection of the insect in multiple HIPVs scenarios (Fig 1). In the first system, adult aphids were used to induce HIPVs. Different numbers of aphids (0, 1, 2, 4, 8 and 16) were transferred to a forty-day-old *A*. *thaliana* plant to produce HIPVs. In the second system, a conidial suspension of *L*. *lecanii* was used to infect the aphids. Conidia of *L*. *lecanii* were cultured with modified Czapek’s medium for 14 d [34], and then the conidia were scraped using an inoculating loop in sterile water containing 0.05% Triton X-80 [43]. The conidial suspension was filtered by cotton in a 20-ml syringe to remove the hyphal debris and then diluted to 5 × 10^7^conidia·mL^-1^ by using a Neubauer hemocytometer with a sterile solution of 0.1% Tween-80 for infection. All suspensions were shaken using a vortex mixer (Lab dancer, IKA®, India Private Limited, Bangalore, Kamataka, INDIA) for 5 min prior to use. Groups of apterous adult aphids were immersed in the conidial suspension for 10 seconds to inoculate the insects with the fungus. As a control, aphids were treated with 0.05% Triton X-80 alone. Following inoculation, all the aphid groups and controls were air dried for approximately 5 min and then separately transferred into a netted bag (50 mesh, 5 cm × 5 cm). The mortality of the aphids in each treatment was recorded every 24 h, and the experiment was replicated thrice.

Four infested plants with the same number (0, 1, 2, 4, 8, or 16) of aphids were transferred into a glass cage (20 cm × 20 cm × 30 cm) to produce different concentrations of HIPVs. A single netted bag containing 50 aphids treated at one of the five fungal concentrations or with Triton X-80 only (control) was hung from the top of the cage **(**Fig 1). Each HIPV-induced plant containing a specific number of aphids was tested as described above. The individual netted bags per concentration were suspended such that they did not come into contact with the leaves of the *A*. *thaliana* plant below them. After a 24-h exposure time inside the cage, all the aphids in the netted bags for each treatment were removed and then transferred to a non-infested *A*. *thaliana* plant. The experiment was conducted at 25°C with a 75% RH and 16 h:8 h L:D photoperiod in the laboratory. Three replicate bags were used per concentration and control to determine the LT_50_ for each treatment. The entire experiment was repeated on four separate occasions.

### The effect of plant HIPVs on the germination of conidia

**Fig 2:**
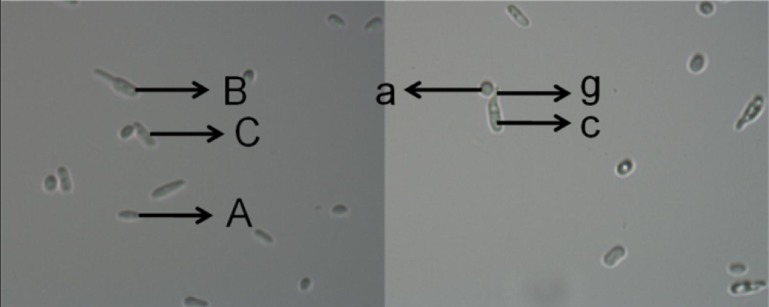
Germination and appressorial formation of *L*. *lecanii* conidia as observed on a concave slide after exposure to different concentrations of HIPVs for 12 hours. i) Non-germinated conidia (A), ii) germinated conidia with no appressoria (B), iii) germinated conidia with appressoria (C), iv) germ tube (g), appressoria (a) and conidia (c).

The experiment using the HIPV system was conducted as described above by placing a different number of healthy aphids on an *A*. *thaliana* plant. However, instead of attaching a netted bag as described above, a 2.5 μL spore suspension (1 × 10^8^ conidia·mL^-1^) prepared in Czapek’s-Dox liquid medium was placed on a concave glass slide (concave at both ends of the slide) according to the protocol of Gonzalez et al. [44]. The sample was allowed to dry, and then the flat side of the slide was attached to the top of the glass cage for each HIPV system. The control slide was attached in a Petri dish (90 mm). The conidial germination of *L*. *lecanii* was determined every 6 h for the duration of each HIPV system exposure time. Every 6 h, slides were quickly removed, and the conidial germination was observed with a light microscope at 1600X magnification (see Fig 2). Three random areas in each concavity in the slide were observed. Three replicates were performed for each serial treatment (aphid number per plant), and the experiment was conducted on four separate occasions.

### The effect of plant HIPVs on the formation of appressorium

The evaluation of the HIPV system was conducted as described above by placing a different number of healthy aphids on an *A*. *thaliana* plant. A 2.5-μL spore suspension (1 × 10^8^ conidia·mL^-1^) prepared in Czapek’s-Dox liquid medium was placed into a concave glass slide and then attached to the top of the glass cage as described above. The control slide was pasted in a petri dish (90 mm). The appressorial formation of *L*. *lecanii* was determined every 6 h for the duration of each HIPV system exposure time as described above (see Fig 2) to obtain the germination rate. Three replicates for each serial treatment (aphid number plant^-1^) were conducted per experiment, and the experiment was conducted on four separate occasions.

### Headspace collection and analysis of volatiles

Headspace collection was conducted from infested *A*. *thaliana* plants during the first 24 h and collected in a system modified from Pineda et al. [45]. Plants were placed into an empty glass jar (12 cm diameter, 30 cm high), and air was forced into the jar after being filtered by activated carbon and silica gel. HIPVs were collected for 6 h by drawing air out of the jars at a rate of 200 to 250 mL∙min^−1^ by a pump through a glass tube filled with 200 mg Tenax TA. Immediately after collection, the HIPVs that were trapped by Tenax TA were eluted by methenyl trichloride and maintained at -20°C.

HIPVs were analyzed with a gas chromatograph (Agilent Technologies 7890B GC System, United States)-mass spectrometer (Agilent Technologies 5977A MSD, United States) (GC-MS) with HP-5 column (30 m × 0.25 mm i.d., 1.0 μm film thickness, Agilent). The GC oven temperature was programmed to hold at 40°C for 2 min followed by a linear thermal gradient of 10°C∙min^−1^. The temperature was then increased to 220°C at 5°C∙min^−1^ and held for 2 min in a column 3 mL∙min^−1^. The column effluent was ionized by electron impact ionization at 70 eV. Mass spectra were acquired by scanning from 35 to 350 m/z with a scan rate of 5.38 scans∙sec^−1^.

Compounds were identified by using the deconvolution software AMDIS (version 2.64, NIST, USA) in combination with NIST 05 and Wiley 7th edition spectral libraries and by comparing their retention indices with those from the literature. The relative quantification of each compound is indicated by its peak area [46].

### Statistical analysis

The sub-lethal time (LT_50_) values were analyzed by comparing the longevity of infected aphids and the controls in the same HIPV system using linear regression. For calculations of the conidial germination, appressorium rates and the peak area of each compound, the data were analyzed using analysis of variance (ANOVA), and the significance of mean differences between the treatments was analyzed using the Least Significance Difference test (α = 0.05) in SPSS. Analysis of correlation between the LT_50_ values of both the germination and the appressorial formation rate of *L*. *lecanii* was also determined. All data analyses were conducted using SPSS (2010) (Release 21.0).

## Results

### Infection of *Lecanicillium lecanii* on aphids as influenced by HIPVs

Laboratory test results of *L*. *lecanii* strain V3450 used against the aphid *L*. *erysimi* exposed to different concentrations of HIPVs for 8 d are summarized in Table 1. The natural control mortality was 10% after 8 days. In treatment 1, 2 and 4, mortalities (55.33± 3.06, 60.00 ± 6.00 and 60.67 ± 3.05, respectively) did not show significant differences with each other and significantly higher than that of treatment 0 (48.00 ± 7.21), but significantly lower than that of treatment 8 (77.33 ± 5.78). The mean values of LT_50_ in all treatments were arranged from low to high, 8 (6.15) < 16 (6.46) < 4 (6.91) <1 (7.09) < 2 (7.11) < 0 (7.87).

**Table 1:** LT_50_ and mortality of infected adult *L*. *erysimi* exposed to HIPVs for 8 days.

Treatments^a^	LT_50_(d)^b^	95% Confidence limits	Regression equation	Mortality^c^
**0**	7.87	7.26~8.76	y = 7.142x - 13.31	48.00 ± 7.21c
**1**	7.09	6.61~7.72	y = 8.865x - 15.64	55.33 ± 3.06b
**2**	7.11	6.60~7.80	y = 8.642x - 13.97	60.00 ± 6.00b
**4**	6.91	6.39~7.61	y = 8.714x - 11.71	60.67 ± 3.05b
**8**	6.15	5.77~6.50	y = 11.12x - 17.90	77.33 ± 5.78a
**16**	6.46	6.03~6.98	y = 10.11x - 15.61	69.33 ± 7.02ab

^a^ Six different HIPV concentrations were produced from 0, 1, 2, 4, 8, and 16 aphids feeding on one plant for each group.

^b^ Median lethal time

^c^ Mean ± standard error (SE) of total mortality in 8 days

### Germination rate of conidia as influenced by HIPVs

**Fig 3:**
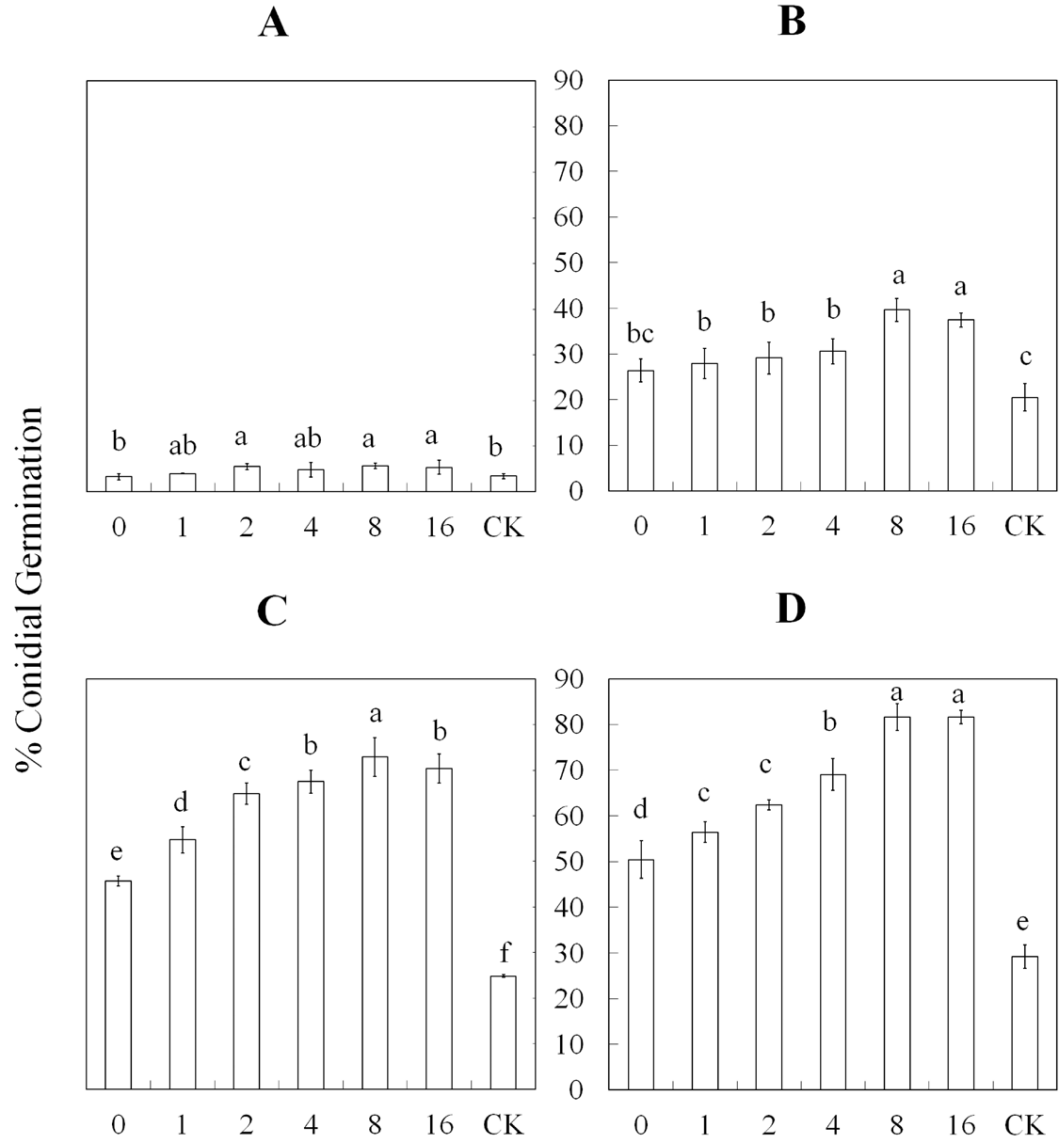
Percent (± SE) germination rate of *L*. *lecanii* conidia after exposure to different concentrations of HIPVs over time. The HIPVs produced from *A*. *thaliana* infested by 0, 1, 2, 4, 8, and 16 aphids, and conidia incubated in clean petri dishes were used as control. The control was abbreviated as CK. Treatments included different exposure times (6, 12, 18 and 24 h) to the induced HIPVs. The germination rate was analyzed after A) 6-, B) 12-, C) 18-, and D) 24-h exposures to the HIPVs. The concentration of *L*. *lecanii* was 1×10^8^ conidia·mL^-1^.

The conidial germination rate after exposure to different concentrations of HIPVs induced by a different number of aphids varied greatly over time (Fig 2, Fig 3 and S1 Table). The germination of conidia in treatment 2, 8, and 16 were increased (*F*_*2*, *6*_ = 2.51; *P* = 0.0728) compared with treatments 0 and 1 and the control (CK) after a 6-h exposure. After a 12-h exposure, treatments 8 and 16 exhibited a significantly increased (*F*_*2*, *6*_ = 14.97; *P* = 0.0001) conidial germination rate compared with the other treatments, including the control and treatment 0 which was slower than all the other treatments. The germination rate of conidia was fastest in treatment 8 and slowest in treatment 0. However, all treatments were significantly faster (*F*_*2*, *6*_ = 126.94; *P* = 0.0001) than the control after an 18-h exposure. Additionally, treatments 4–16 were not significantly different, but they were significantly faster than treatments 0–2. Treatments 8 and 16 exhibited significantly increased (*F*_*2*, *6*_ = 130.04; *P* = 0.0001) germination rates compared with the other treatments and the control after a 24-h exposure to the HIPVs. In general, the germination rates of the conidia after a 12-h exposure to the different concentrations of HIPVs were faster than the control, which did not include aphids.

### Appressorium formation as influenced by HIPVs

**Fig 4:**
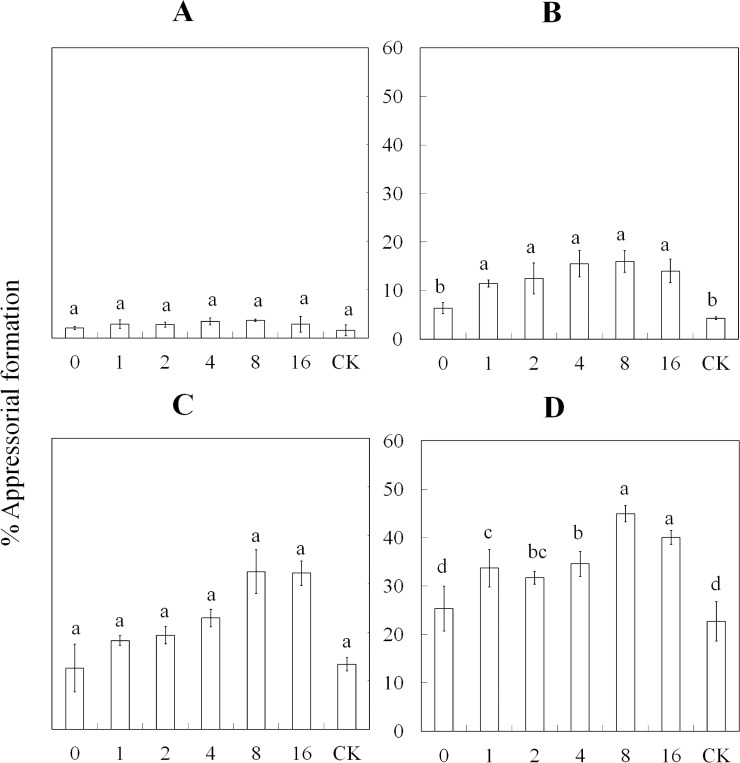
Percent (± SE) appressorial formation of *L*. *lecanii* conidia after exposure to different concentrations of HIPVs over time. The HIPVs produced from *A*. *thaliana* infested by 0, 1, 2, 4, 8, and 16 aphids, and conidia incubated in clean petri dishes were used as control. The control was abbreviated as CK. The treatments had different exposure times (6, 12, 18 and 24 h) to the HIPVs. The appressorial rate of formation was analyzed after A) 6; B) 12; C) 18; D) or 24 h exposure. The supernatant concentration of *L*. *lecanii* was diluted to 1×10^8^ conidia·mL^-1^.

The results of the percent appressorial formation with exposure to HIPVs induced by the different numbers of aphids are presented in Fig 2, Fig 4 and S2 Table. After a 6-h exposure to the HIPVs, no significant differences were noted among all treatments. In treatments 1–16, the percent appressorial formation was significantly increased (*F*_*2*, *6*_ = 9.29; *P* = 0.0003) compared with treatment 0 and the control after a 12-h exposure. At 18 h post-exposure, the appressorium developed faster (*F*_*2*, *6*_ = 27.29; *P* = 0.0001) in treatments 8 and 16 compared with treatments 1–4, which all exhibited similar percent formation but were faster than treatment 0 and the control. Treatments 8–16 were similar, and the percent formation of the appressoria was significantly increased (*F*_*2*, *6*_ = 18.44; *P* = 0.0001) compared with the other treatments after being exposed to HIPVs for 24 h. In addition, the percent appressorial formation was increased in treatments 1–4 compared with treatments 0 and the control; only treatments 0 and the control were similar and had the lowest percent formation compared with the other treatments. In general, after an 18-h exposure to the HIPVs, treatments 8–16 had the highest percentage of appressoria formed compared with the other treatments.

### Aphid densities modified the profile of HIPVs from *A*. *thaliana*

Twelve major compounds were detected in the headspace of *A*. *thaliana* that were fed on by aphids. The results indicate that the area peaks of decan-3-ol, benzaldehyde, phenylacetaldehyde, 4-methylpentyl isothiocyanate and α-terpineol could not be detected in treatment 0, whereas 1-octen-3-ol, 6-methyl hept-5-en-2-one, 2,2-dimethyl-1-butanol, limonene and menthol did not exhibit significant differences among the treatments (Table 2 and S1A:, S1B: S1C: S1D: S1E: and S1F Fig). Decan-3-ol was only detected in treatment 16, and the peak area was 119052.67 ± 19982.46 (*F*_*2*, *0*_ = 106.48). The peak area of the benzaldehyde detected in treatment 8, was significantly increased compared with treatment 2 and 4 (*F*_*2*, *3*_ = 5.73; *P* = 0.0216). The peak area of phenylacetaldehyde did not exhibit significant differences in the treatments in which phenylacetaldehyde was detected (*F*_*2*, *4*_ = 2.328; *P* = 0.127). The peak area of 4-methylpentyl isothiocyanate in treatment 8 (*F*_*2*, *3*_ = 11.96; *P* = 0.0025) was significantly increased compared with the other treatments. α-Terpineol was only detected in treatments 8 and 16, and no significant difference was noted between the peak areas (*F*_*2*, *1*_ = 0.48; *P* = 0.53). The peak area of 4-methyl-1-penten-3-ol in treatment 0 (*F*_*2*, *5*_ = 28.28; *P* = 0.001) was significantly increased compared with the others treatments, whereas the area of 5-methyl-3-hexanol in treatment 8 (*F*_*2*, *5*_ = 11.55; *P* = 0.001) was significantly increased compared with the other treatments.

**Table 2:** Major compounds emitted from *A*. *thaliana* infested by different densities of *L*. *erysimi* in 24 hours. Data are reported as the mean peak area ± standard deviation (SD) of values of three replicates. Means followed by different letters are significantly different (P < 0.05, one-way ANOVA, Least Significance Difference test), ‘nd’ means undetected.

Compounds	Insect density					
	0	1	2	4	8	16
**Decan-3-ol**	nd	nd	nd	nd	nd	119052.67±19982.46
**Benzaldehyde**	nd	nd	14182.33±4596.23b	13530.67±3974.81b	32993.67±11120.47a	26550.33±5557.76ab
**Phenylacetaldehyde**	nd	9443.00±2273.06a	13347.00±5693.95a	5033.66±3694.87a	8696.33±1693.89a	8128.67±1823.38a
**4-methylpentyl isothiocyanate**	nd	nd	4973.00±2469.20b	27511.33±15142.41b	389790.33±161978.90a	129853.00±69043.61b
**α-Terpineol**	nd	nd	nd	nd	44969.33±25078.87a	34517.67±7737.01a
**6-Methyl hept-5-en-2-one**	566773.67±178447.29a	492802.33±195227.37a	564150.33±223597.04a	474091.00±168735.92a	459241.00±128623.00a	319327.33±153739.46a
**1-Octen-3-ol**	35962±17399.88a	40329.33±26260.96a	43139±16189.12a	48815.33±17158.75a	49225.33±29073.80a	41623.67±22858.13a
**2,2-Dimethyl-1-butanol**	88603.33±32255.92a	70132±27931.15a	73059.67±16764.1a	83442±9951.98a	55480.67±22865.95a	66119±29749.43a
**Limonene**	5771.67±2402.66a	5181±2524.47a	4937.33±3512.66a	5743±2828.1a	5373±3179.86a	11338±7671.69a
**Menthol**	5113.67±2105.34a	8152±3329.9538a	3409.67±1970.45a	6697.33±3318.06a	39310±1365.90a	5405.00±3648.37a
**4-Methyl-1-penten-3-ol**	3020657.67±636263.76a	557273.00±188672.61b	582240±126145.6903b	624548.00±253567.94b	582294.00±142243.97b	547386.00±303443.08b
**5-Methyl-3-hexanol**	5109.33±3700.81c	14622.33±3945.65bc	27295.00±17257.87bc	61919.33±18132.80bc	294275.33±131252.95a	117880.33±28869.94b

### Correlation analysis for LT50 and conidia performance

The correlation between pathogenicity and conidial performance of *V*. *lecanii* as influenced by HIPVs is summarized in Table 3. The correlation coefficients for both biological processes after exposure to HIPVs were less than -0.5, indicating a significant negative correlation trend over time. The highest negative correlation coefficient between LT_50_ values and the percent conidial germination was -0.90 after an 18-h exposure to the HIPVs. However, between the LT_50_ and the formation of the appressorium, the correlation coefficient was much higher (-96) after only a 12-h exposure to the HIPVs.

**Table 3:** Analysis of correlation between the LT_50_ values and performance of conidia as influenced by HIPVs.

	Correlation analysis for LT_50_ and germination rate				Correlation analysis for LT_50_ and % appressorial formation			
	6 h	12 h	18 h	24 h	6 h	12 h	18 h	24 h
**Correlation coefficient**	-0.69	-0.92	-0.97	-0.95	-0.86	-0.92	-0.97	-0.99

## Discussion

The emission of HIPVs are one of the strategies that plants utilize to relieve herbivorous stress by attracting insect enemies and giving warning to nearby plants [47–52]. HIPVs can act as a bridge in the tri-trophic system of the plant-herbivore-predator community [26]. In addition to predators, some microorganisms, which are important natural enemies, can have a positive impact by infecting herbivores that are feeding on the plant. Although some evidence has suggested that the performance and pathogenicity of microorganisms is impacted by the emission of VOCs from plants [23–25], the interaction of entomopathogenic fungi and plants in a tri-trophic system of plant-herbivore-entomopathogenic fungi has not been reported. In our experiment, HIPVs were induced by aphids feeding on a plant, and the volatiles were exposed to contaminated adult aphids that acquired conidia of entomopathogenic fungi. Our results indicated that the percent germination rate and appressorial formation of *L*. *lecanii* against *L*. *erysimi* within the HIPV induced system was significantly increased compared with that without HIPVs. Significant negative correlations were noted between LT_50_ values and both the conidial germination rate and appressorium formation of *L*. *lecanii*, suggesting that HIPVs can increase the pathogenicity of entomopathogenic fungi by promoting conidial germination or appressorial formation [30,53]. Benzaldehyde and 4-methylpentyl isothiocyanate are probably the chemicals that encourage entomopathogenic fungi according to the headspace analysis.

Insects are the key factor that induce HIPVs; therefore, the composition and emission rate of HIPVs might be influenced by the number, species, age, sex, and physical condition of each insect. Rodriguez-Saona et al. [22] observed that as the perennial shrub *Vaccinium corymbosum* became gradually infested by gypsy moth (*Lymantria dispar*) caterpillars over time, the plant VOC emission rate also increased concomitantly. Our results revealed that the exposure of HIPVs emitted from *A*. *thaliana* to *L*. *lecanii* increased with the subsequent increase in the number of aphids on the plant. In addition, it appears that the concentration of HIPVs did not increase when the number of aphids reached the critical value or asymptote of ≥ 8 aphids per plant for the optimum threshold production of VOCs from this plant. Therefore, it could be hypothesized that the emission rate of HIPVs is proportional to the number of insects present on the plant until the critical number of insect density is reached. Decan-3-ol was detected only in the headspace in the treatment with 16 aphids per plant, and the amount of 4-methylpentyl isothiocyanate in the treatment with 8 aphids per plant was significantly higher than the other treatments. It appears that the emergence of decan-3-ol and/or the decrease of 4-methylpentyl isothiocyanate led to the down-regulation of the activity of the HIPVs inducing the conidial germination of entomopathogenic fungi.

The appearance of an optimum aphid density that is proportional to the emission rate of induced HIPVs may be explained by some physiological processes a plant may employ in response to an ever increasing population of feeding insects. Plants may down-regulate the emission rate of HIPVs or modify the constitution of HIPVs when the critical insect number has reached the VOC production threshold. In addition to the density of insects, the reduction in HIPV emissions may be regulated when a sufficient number of symbiotic bacteria from the oral secretions or honeydew production from these insects deceive the plant into reducing its defensive production of VOCs [54,55]. These hypotheses warrant more research to be confirmed. The emission rate and constitution of HIPVs induced by the different number of insects per plant as well as the mechanism by which conidia are influenced by HIPVs was not investigated in this study.

In conclusion, we provided direct evidence that HIPVs derived from insects functioning as inductors promoted the performance and pathogenicity of conidia of entomopathogenic fungi. In addition, a critical optimal threshold level was determined for aphid density that produced the optimum amount of HIPVs to promote the highest conidial performance of the entomopathogenic fungi. Our results provide important clues that HIPVs play a significant role and provide a bridge in the plant-herbivore-entomopathogenic fungi tri-trophic system. This study also adds a unique dimension to our understanding of how plants provide feedback to their ‘bodyguards’.

## Supporting Information

S1 Table Percent (± SE) germination rate of *L*. *lecanii* conidia after exposure to different concentrations of HIPVs over time. (DOCX)

S2 Table Percent (± SE) appressorial formation of *L*. *lecanii* conidia after exposure to different concentrations of HIPVs over time. (DOCX)

S1 Fig A: (Mass spectrogram of the headspace collected from 0-aphid-induced Arabidopsis). B: (Mass spectrogram of the headspace collected from 1-aphid-induced Arabidopsis). C: (Mass spectrogram of the headspace collected from 2-aphids-induced Arabidopsis). D: (Mass spectrogram of the headspace collected from 4-aphids-induced Arabidopsis). E: (Mass spectrogram of the headspace collected from 8-aphids-induced Arabidopsis). F: (Mass spectrogram of the headspace collected from 16-aphids-induced Arabidopsis).
(ZIP)
