# Supplementary material for: Volatiles from Plants Induced by Multiple Aphid Attacks Promote Conidial Performance of Lecanicillium lecanii
Source: PLoS One. 2016 Mar 21;11(3):e0151844. doi: 10.1371/journal.pone.0151844 (PMC4801321; doi:10.1371/journal.pone.0151844)
Supplement: S1 Table — (DOCX) [file pone.0151844.s001.docx]

| Treatments | (Germination rate ± SE)% | | | |
| --- | --- | --- | --- | --- |
|  | 6h | 12h | 18h | 24h |
| 0 | 3.36±0.67b | 26.34±2.51bc | 45.74±1.1e | 50.42±4.12d |
| 1 | 4.03±0.08ab | 27.88±3.29b | 54.75±2.87d | 56.43±2.28c |
| 2 | 5.55±0.69a | 29.11±3.53b | 64.8±2.32c | 62.38±1.12c |
| 4 | 4.85±1.59ab | 30.57±2.77b | 67.52±2.55b | 69.03±3.48b |
| 8 | 5.71±0.6a | 39.64±2.49a | 72.89±4.26a | 81.62±2.92a |
| 16 | 5.37±1.57a | 37.43±1.59a | 70.35±3.2b | 81.61±1.46a |
| Control | 3.47±0.53b | 20.49±2.96c | 24.86±0.29f | 29.22±2.56e |

**S1 Table. Percent (± SE) germination rate of *L. lecanii* conidia after exposure to different concentrations of HIPVs over time.**
