# Supplementary material for: Volatiles from Plants Induced by Multiple Aphid Attacks Promote Conidial Performance of Lecanicillium lecanii
Source: PLoS One. 2016 Mar 21;11(3):e0151844. doi: 10.1371/journal.pone.0151844 (PMC4801321; doi:10.1371/journal.pone.0151844)
Supplement: S2 Table — (DOCX) [file pone.0151844.s002.docx]

**S2 Table. Percent (± SE) appressorial formation of *L. lecanii* conidia after exposure to different concentrations of HIPVs over time**.

| Treatments | (Appressoral formantion rate ± SE)% | | | |
| --- | --- | --- | --- | --- |
|  | 6h | 12h | 18h | 24h |
| 0 | 2.08±0.31a | 6.4±1.15b | 12.61±4.89d | 25.31±4.64d |
| 1 | 2.86±0.87a | 11.47±0.7a | 18.27±1.02c | 33.71±3.87c |
| 2 | 2.76±0.52a | 12.51±3.17a | 19.39±1.82bc | 31.71±1.32c |
| 4 | 3.42±0.74a | 15.51±2.69a | 22.92±1.77b | 34.6±2.6bc |
| 8 | 3.68±0.25a | 16.01±2.25a | 32.48±4.53a | 44.93±1.68a |
| 16 | 2.83±1.68a | 14.04±2.43a | 32.17±2.51a | 40.03±1.46b |
| Control | 1.59±1.1a | 4.31±0.27b | 13.39±1.38d | 22.68±4.11d |
