# Supplementary figures and images for: Volatiles from Plants Induced by Multiple Aphid Attacks Promote Conidial Performance of Lecanicillium lecanii
Source: PLoS One. 2016 Mar 21;11(3):e0151844. doi: 10.1371/journal.pone.0151844 (PMC4801321; doi:10.1371/journal.pone.0151844)

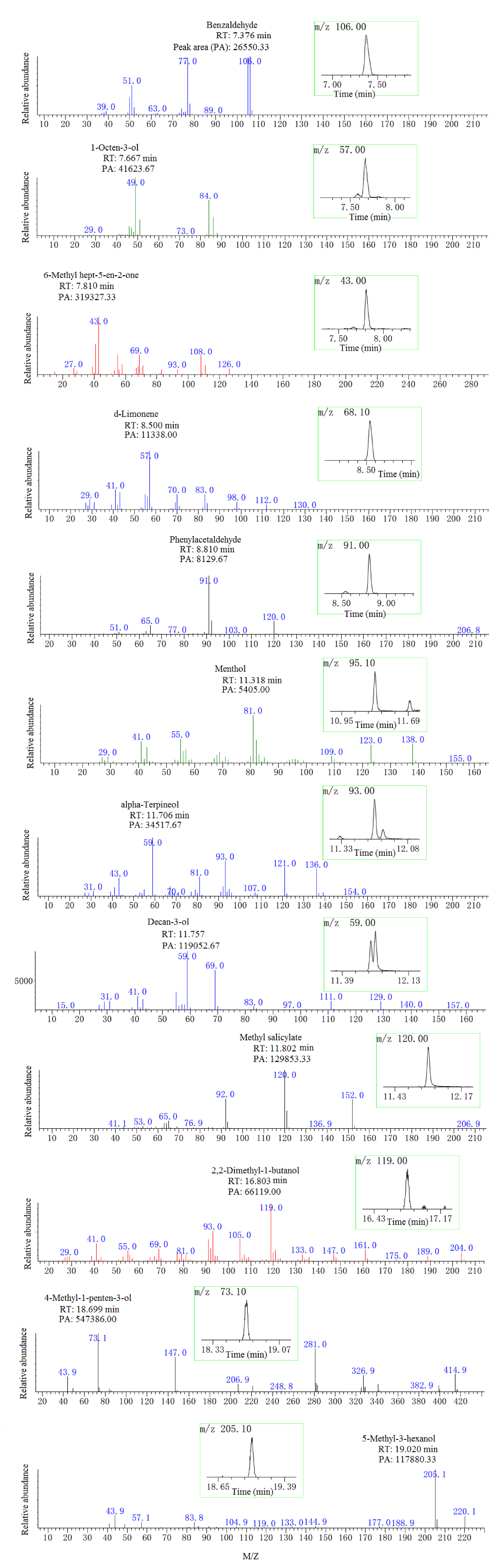

Supplement: S1 Fig — A: (Mass spectrogram of the headspace collected from 0-aphid-induced Arabidopsis). B: (Mass spectrogram of the headspace collected from 1-aphid-induced Arabidopsis). C: (Mass spectrogram of the headspace collected from 2-aphids-induced Arabidopsis). D: (Mass spectrogram of the headspace collected from 4-aphids-induced Arabidopsis). E: (Mass spectrogram of the headspace collected from 8-aphids-induced Arabidopsis). F: (Mass spectrogram of the headspace collected from 16-aphids-induced Arabidopsis). (ZIP) [file pone.0151844.s003.zip › S1 Fig. F (Mass spectrogram of the headspace collected from 16-aphids-induced Arabidopsis).tif]

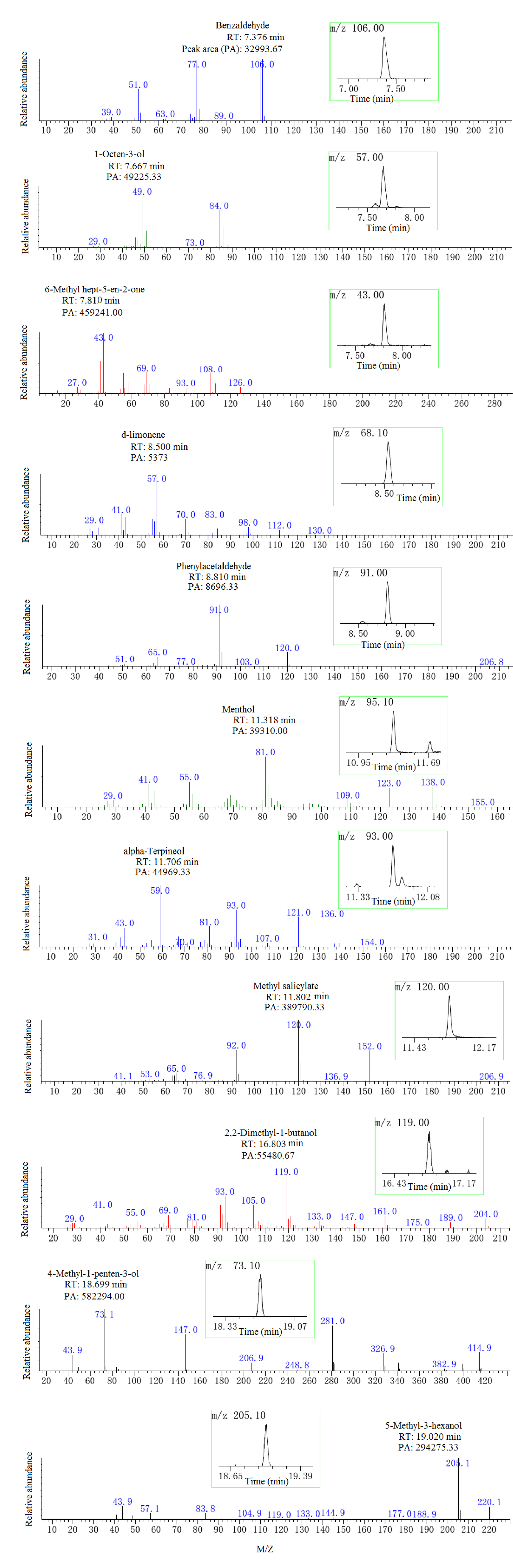

Supplement: S1 Fig — A: (Mass spectrogram of the headspace collected from 0-aphid-induced Arabidopsis). B: (Mass spectrogram of the headspace collected from 1-aphid-induced Arabidopsis). C: (Mass spectrogram of the headspace collected from 2-aphids-induced Arabidopsis). D: (Mass spectrogram of the headspace collected from 4-aphids-induced Arabidopsis). E: (Mass spectrogram of the headspace collected from 8-aphids-induced Arabidopsis). F: (Mass spectrogram of the headspace collected from 16-aphids-induced Arabidopsis). (ZIP) [file pone.0151844.s003.zip › S1 Fig. E (Mass spectrogram of the headspace collected from 8-aphids-induced Arabidopsis).tif]

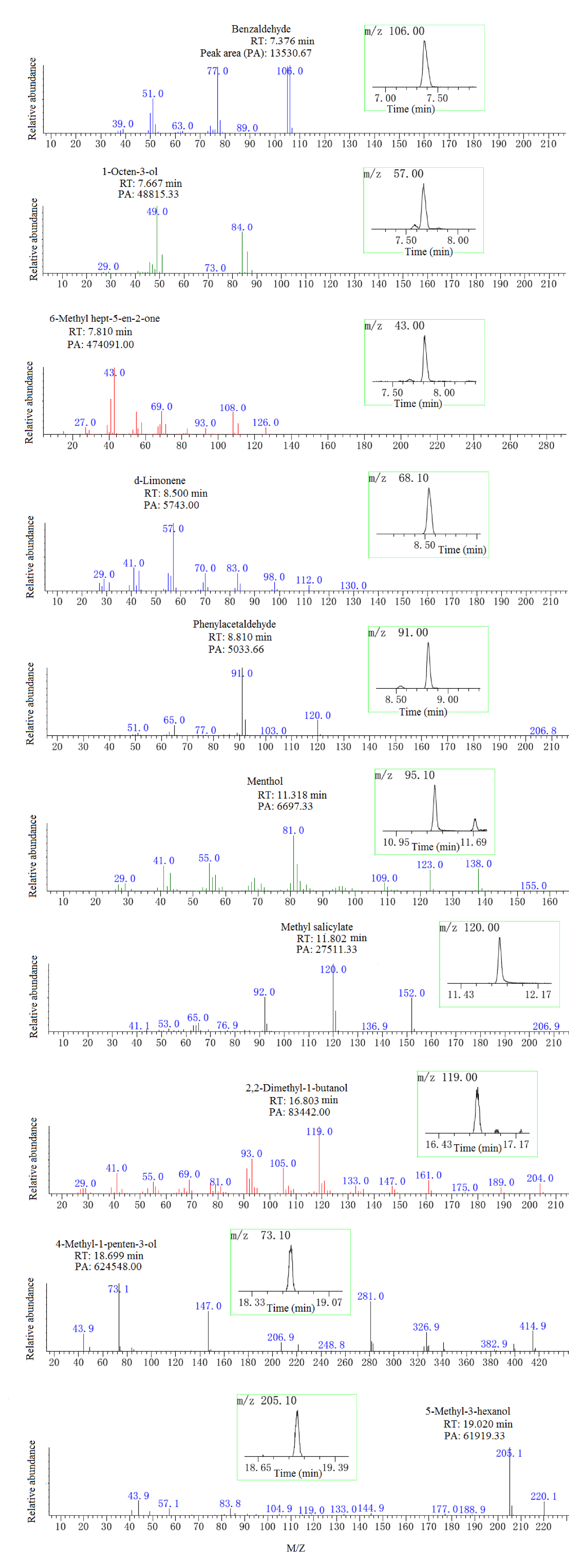

Supplement: S1 Fig — A: (Mass spectrogram of the headspace collected from 0-aphid-induced Arabidopsis). B: (Mass spectrogram of the headspace collected from 1-aphid-induced Arabidopsis). C: (Mass spectrogram of the headspace collected from 2-aphids-induced Arabidopsis). D: (Mass spectrogram of the headspace collected from 4-aphids-induced Arabidopsis). E: (Mass spectrogram of the headspace collected from 8-aphids-induced Arabidopsis). F: (Mass spectrogram of the headspace collected from 16-aphids-induced Arabidopsis). (ZIP) [file pone.0151844.s003.zip › S1 Fig. D (Mass spectrogram of the headspace collected from 4-aphids-induced Arabidopsis).tif]

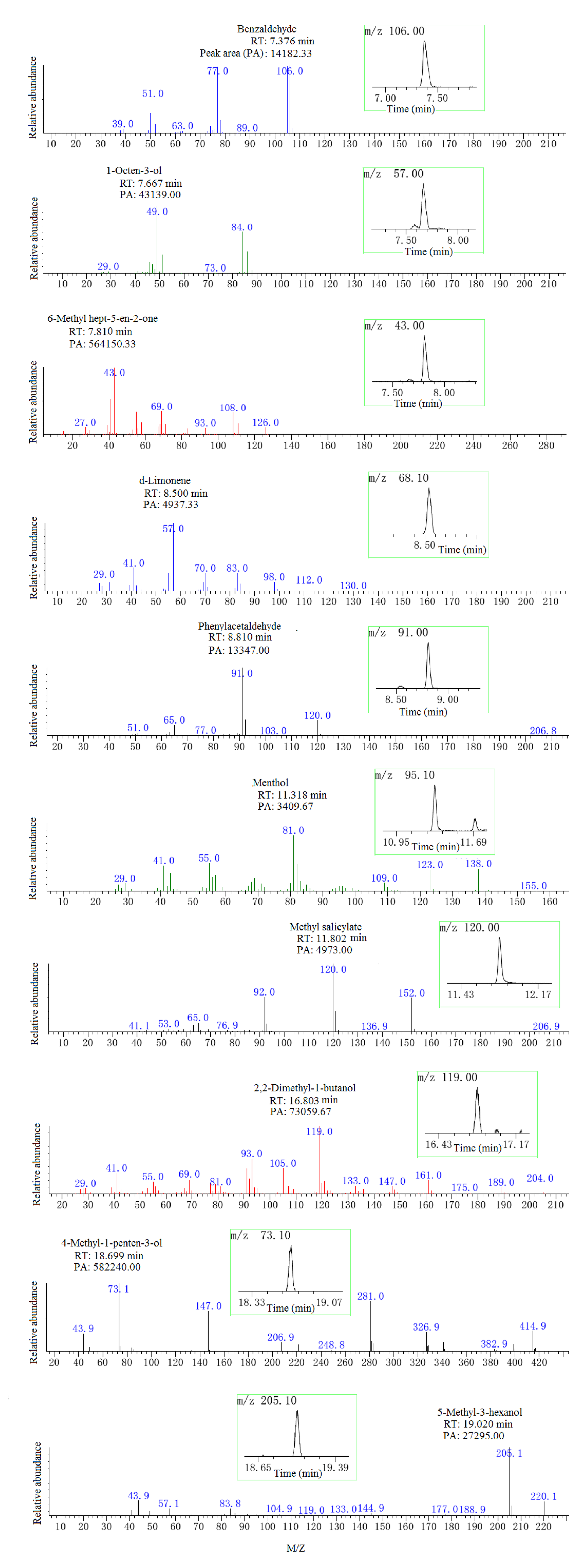

Supplement: S1 Fig — A: (Mass spectrogram of the headspace collected from 0-aphid-induced Arabidopsis). B: (Mass spectrogram of the headspace collected from 1-aphid-induced Arabidopsis). C: (Mass spectrogram of the headspace collected from 2-aphids-induced Arabidopsis). D: (Mass spectrogram of the headspace collected from 4-aphids-induced Arabidopsis). E: (Mass spectrogram of the headspace collected from 8-aphids-induced Arabidopsis). F: (Mass spectrogram of the headspace collected from 16-aphids-induced Arabidopsis). (ZIP) [file pone.0151844.s003.zip › S1 Fig. C (Mass spectrogram of the headspace collected from 2-aphids-induced Arabidopsis).tif]

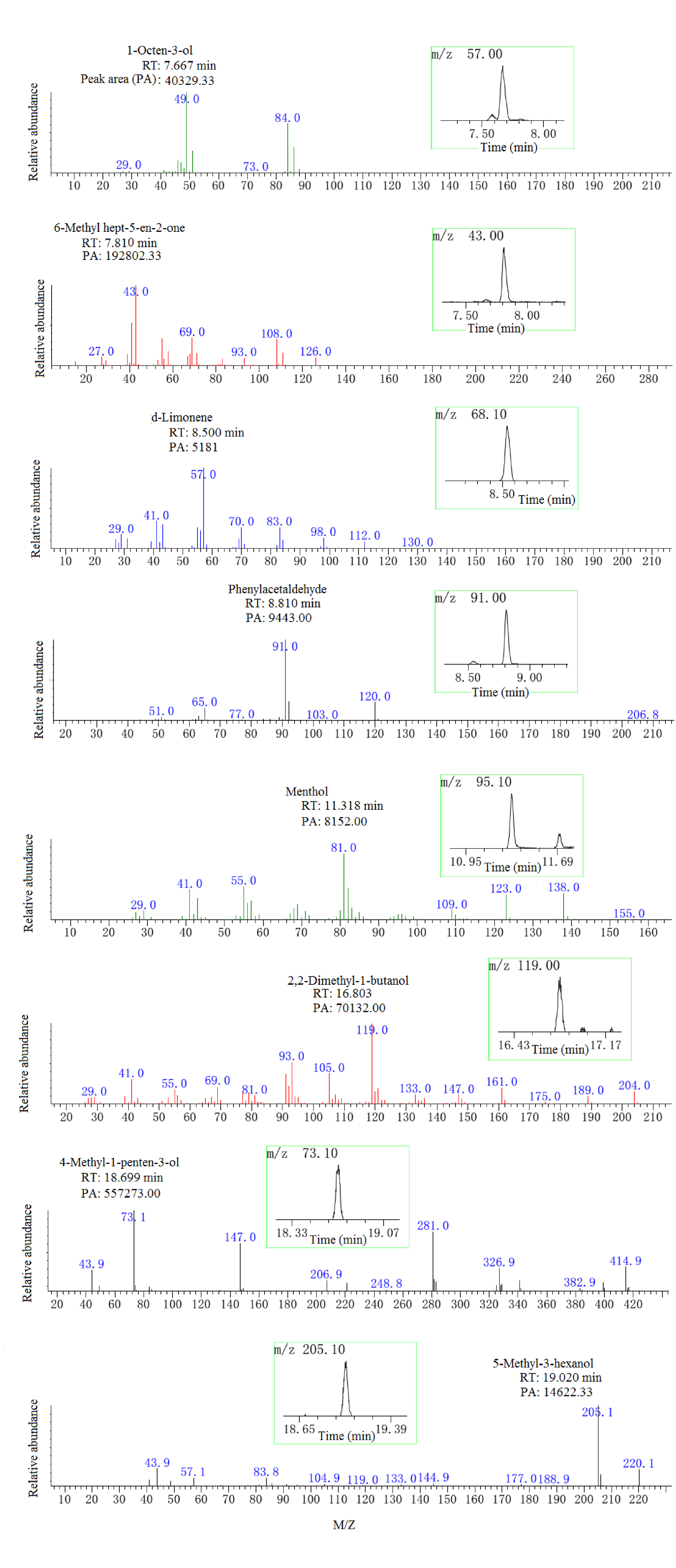

Supplement: S1 Fig — A: (Mass spectrogram of the headspace collected from 0-aphid-induced Arabidopsis). B: (Mass spectrogram of the headspace collected from 1-aphid-induced Arabidopsis). C: (Mass spectrogram of the headspace collected from 2-aphids-induced Arabidopsis). D: (Mass spectrogram of the headspace collected from 4-aphids-induced Arabidopsis). E: (Mass spectrogram of the headspace collected from 8-aphids-induced Arabidopsis). F: (Mass spectrogram of the headspace collected from 16-aphids-induced Arabidopsis). (ZIP) [file pone.0151844.s003.zip › S1 Fig. B (Mass spectrogram of the headspace collected from 1-aphid-induced Arabidopsis).tif]

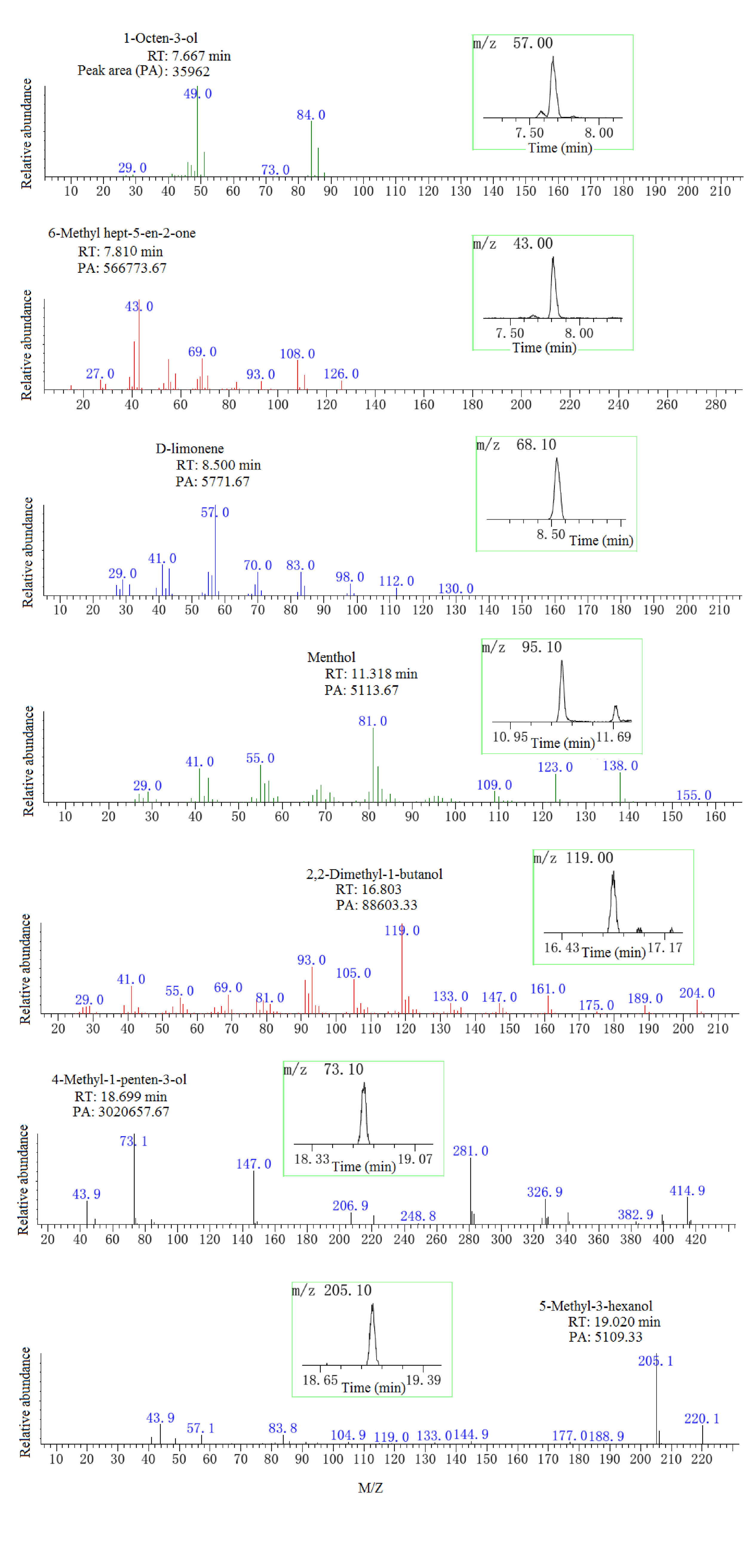

Supplement: S1 Fig — A: (Mass spectrogram of the headspace collected from 0-aphid-induced Arabidopsis). B: (Mass spectrogram of the headspace collected from 1-aphid-induced Arabidopsis). C: (Mass spectrogram of the headspace collected from 2-aphids-induced Arabidopsis). D: (Mass spectrogram of the headspace collected from 4-aphids-induced Arabidopsis). E: (Mass spectrogram of the headspace collected from 8-aphids-induced Arabidopsis). F: (Mass spectrogram of the headspace collected from 16-aphids-induced Arabidopsis). (ZIP) [file pone.0151844.s003.zip › S1 Fig. A (Mass spectrogram of the headspace collected from 0-aphid-induced Arabidopsis).tif]
